# Female Physicians

**Published:** 1867-06

**Authors:** 


FEMALE PHYSICIANS.

Dr. Atlee, of Philadelphia, offered the following:

*Whereas*, The subject of female medical education is exciting attention, and regularly educated female physicians have established themselves as practitioners of medicine; and

*Whereas*, Female Medical Colleges, embracing all branches taught in other colleges, and all the conditions for graduation exist in the United States for the separate education of females; and

*Whereas*, It is important that the standard of education, and the observance of the code of medical ethics should be fostered and maintained by this Association; therefore,

*Resolved*, That the American Medical Association recognizes well educated female physicians by the same laws that govern its own members.

Dr. Bowditch arose to a point of order, and reminded the President that the Association had postponed the order of the day for five minutes to Dr. Storer's question of privilege. He claimed that the five minutes had expired, and he moved to lay the resolutions on the table, which motion prevailed without a negative vote.

